# Mechanism and future prospect of treatment of facial paralysis caused by herpes zoster virus infection with acupuncture combined with medicine: A review

**DOI:** 10.1097/MD.0000000000039652

**Published:** 2024-09-20

**Authors:** Ruiqian Guan, Zhibo Hong, Limin Pan, Yusu Wang, Yeyao Li

**Affiliations:** a Heilongjiang University of Chinese Medicine/Second Affiliated Hospital of Heilongjiang University of Chinese Medicine, Harbin City, Heilongjiang Province, China; b Heilongjiang University of Chinese Medicine, Harbin City, Heilongjiang Province, China; c Heilongjiang University of Chinese Medicine/The First Affiliated Hospital of Heilongjiang University of Chinese Medicine, Harbin City, Heilongjiang Province, China.

**Keywords:** acupuncture and moxibustion, facial paralysis, herpes zoster, hunt syndrome, traditional Chinese medicine combination of acupuncture and medicine

## Abstract

Herpes zoster virus infectious facial paralysis is caused by the reactivation and replication of varicella-zoster virus, which leads to herpetic inflammatory lesions, resulting in peripheral facial paralysis associated with herpes rash in the auricle and external ear, and vestibular cochlear dysfunction. It is also known as Ramsey-Hunter syndrome (Hunt syndrome). Facial paralysis caused by herpes zoster is difficult to cure due to its easy loss of treatment and mistreatment. Cause a greater burden on the patient’s body and mind. However, the treatment of Western medicine has lagged behind and there are many adverse reactions, which cannot be completely cured, and new alternatives are urgently needed. This article briefly reviews the advantages and disadvantages of modern medical treatment of Hunt syndrome. This paper expounds the unique ideas of traditional Chinese medicine in the treatment of Hunt syndrome from the perspectives of antiviral, antibacterial, improving blood circulation, protecting cardiovascular, cerebrovascular, and nerve. This article discusses the superiority of traditional Chinese medicine in the treatment of Hunt syndrome from 2 aspects of Chinese medicine therapy and acupuncture therapy, and points out the feasibility of combined treatment of acupuncture and traditional Chinese medicine. So as to provide a new treatment for Hunt syndrome.

## 1. Introduction

Herpes zoster virus-infected facial paralysis is caused by reactivation and replication of varicella-zoster virus latent in the genicular ganglion and facial nerve (the 7th cranial nerve), which leads to herpetic inflammation from the ganglion to the adjacent affected ganglion and its related dermatomes, thus causing peripheral facial paralysis related to herpes rash of auricle and external ear. And vestibulocochlear dysfunction, also known as Ramsey-Hunter syndrome (Hunt syndrome).^[1,2]^ Hunt syndrome accounts for approximately 12% of cases of acute peripheral facial paralysis, and herpes zoster infective facial paralysis accounts for 7% of these cases,^[3,4]^ and has a high incidence in different age groups. According to statistics, this disease accounts for 16% of all causes of unilateral facial paralysis in children and 18% in adults,^[5]^ and is the second most common cause of nontraumatic facial neuropathy after Bell’s palsy^[6]^ and less likely to recover completely,^[7]^ with only a 70% chance of return to normal or near-normal function. Patients presented early in the course of the illness with symptoms of ear pain, herpes, nonspecific headache, fever, and fatigue for 1 to 3 days before facial paralysis,^[8]^ so in the early stage, there is a greater probability of being diagnosed as a cold and mistreated, leading to the occurrence of facial paralysis. Facial paralysis is characterized by weakness of the affected facial muscles, manifested by reduced forehead wrinkles, inability to close the eyes, and drooping corners of the mouth,^[9]^ and concomitant symptoms such as saliva dripping from the corners of the mouth and blurred vision.^[10]^ It can be seen that the disease is difficult to diagnose and the prognosis is poor. The most reliable and sensitive method to confirm that diagnosis of the disease is to assess the presence of viral deoxyribose nucleic acid in the invol tissue and vesicular exudates by polymerase chain reaction,^[11]^ but the clinical popularization rate is low and the clinical diagnostic value is low.

Corticosteroids, antiviral drugs, vasodilators, neurotrophic drugs, and symptomatic treatment are the main modern medical treatment options,^[12,13]^ but the treatment effect is single and not ideal, especially the cure rate of patients with late facial paralysis is very low.^[14]^

Compared with the single treatment method of modern medicine, traditional Chinese medicine is widely popular in Southeast Asia because of its wide variety of drugs, less side effects and excellent therapeutic effects. Although it is not as widely spread as acupuncture, it is still a rare choice. Acupuncture therapy is to make the body respond positively to the external stimulation generated by acupuncture and moxibustion on the human body. These reactions have a therapeutic effect on the corresponding diseases. At the same time, acupuncture is widely accepted by the people of the world because of its nontoxic side effects, fast and efficient characteristics. However, the curative effect of single acupuncture therapy or traditional Chinese medicine therapy on chronic diseases is relatively weak.

Therefore, the author hopes to explore the therapeutic mechanism of traditional Chinese medicine by summarizing the methods and schemes of traditional Chinese medicine in the treatment of this disease in recent years. Discuss the feasibility of acupuncture combined with medicine therapy and summarize its preliminary results. So as to provide more treatment for Hunt syndrome.

## 2. The mechanism and common acupoints of moxibustion in the treatment of facial paralysis caused by herpes zoster virus infection

Herpes zoster virus infective facial paralysis belongs to the category of peripheral facial paralysis, and traditional Chinese medicine treatment of this disease has a very good effect. Facial paralysis belongs to the category of disease of meridians and tendons, the pathogenesis of which is due to the deficiency of vital qi, insufficiency of meridians and collaterals, and the invasion of exogenous pathogens (wind, cold, etc), resulting in the blockage of qi and blood in meridians, tendons and collaterals, and the occurrence of facial paralysis symptoms such as mouth and eye deviation.^[15]^

Acupuncture therapy mainly includes acupuncture at acupoints such as Zanzhu, Yangbai, Taiyang, Yifeng, Qianzheng, Dicang, Jiache, Xiaguan, Chengjiang, Yingxiang, and Sibai,^[16,17]^ combined with moxibustion and massage, can smooth the meridians and collaterals, accelerate the expulsion of exogenous pathogens, and improve facial symptoms.^[18]^ In addition, the above facial acupoints combined with Hegu acupoint can further dredge facial meridian qi; Yifeng, Tinggong, and Waiguan can clear liver and purge fire, relieve depression and dredge collaterals, and improve ear symptoms. Acupuncture at Zusanli can tonify the deficiency of the human body and improve physical fitness.^[19,20]^ Acupuncture therapy is not limited to traditional filiform needling, but also very common in electroacupuncture, fire needle, acupoint injection and other ways.

## 3. Common methods of acupuncture treatment of herpes zoster virus infectious facial paralysis

In the acute stage of the disease, distal acupoints (such as the sternal and clavicular segments of the sternocleidomastoid muscle) are mainly selected in order to reduce the risk of facial nerve edema. The reason why distal acupoints are selected for the sternocleidomastoid muscle is that the tension on both sides of the muscle is asymmetric.^[21]^ In addition, the treatment principle of traditional Chinese medicine in acute stage is to quickly expel pathogenic factors, purge heat and relieve pain,^[22]^ which can be treated with local periauricular acupuncture bloodletting or fire needle, can not only replace antiviral and hormone drugs to reduce toxic and side effects, but also regulate local inflammatory factors to reduce ear pain reaction and promote local blood circulation to accelerate nerve recovery.^[23]^ In the convalescent and sequelae period, facial acupoints were mainly selected to restore facial function by dredging meridians and collaterals and tonifying qi and blood.^[24]^ At this time, special needling techniques are required, such as lifting and pulling of the needle, intermittent wave electroacupuncture, etc, to prevent muscle atrophy and stimulate muscle activity.^[25]^ The face is the part of the 3 Yang meridian tendons of the hand, so it is also a treatment direction to treat it through the meridian tendon theory, so the meridian tendon needling method emerges as the times require. The specific therapy is to perform row needling, shallow needling, penetration needling, and surrounding needling on the local focus or Ashi acupoint to dredge and nourish the meridian tendons, so as to improve the facial symptoms.^[26]^

From the above, we can see that acupuncture and moxibustion therapy is mainly based on local acupoint selection, the purpose is to improve local symptoms and reduce the harm of sequelae, but the sequelae of this disease are serious and need long-term treatment, only long-term persistent acupuncture treatment can gradually improve symptoms and achieve the ultimate goal of improving the quality of life of patients.

## 4. Common drugs and mechanism of action for the treatment of herpes zoster virus infectious facial paralysis

There are many kinds of Chinese herbal medicines, among which Scorpio, Bombyx Batryticatus, Rhizoma Chuanxiong, Rhizoma Typhonii, Radix Angelicae Sinensis, Radix Saposhnikoviae, Radix Astragali, Radix Angelicae Dahuricae, Scolopendra, and Radix Paeoniae Alba are commonly used for the treatment of facial paralysis,^[27,28]^ which is widely used in the treatment of various types of facial paralysis. Take the above drugs as an example, their mechanism of action is as follows.

The active components of scorpion can activate phosphatidylinositol 3 kinase pathway/protein kinase B pathway signaling pathway to promote the release of nitric oxide from vascular endothelial cells and regulate the tension and permeability of blood vessels, and have a very good inhibitory effect on spasm^[29]^; the active constituents of Bombyx Batryticatus have antihyperglycemic, antiobesity, antiviral, anticonvulsant, anticoagulant, antithrombotic, and anti-tumor properties.^[30]^ The polysaccharide of Ligusticum chuanxiong Hort., the active ingredient of Ligusticum chuanxiong Hort., has a variety of activities, such as antioxidation, immune promotion, anti-tumor, antibacterial, and so on, which makes its application extremely extensive.^[31]^ In addition, Rhizoma Typhonii cerebroside, the active ingredient of Rhizoma Typhonii, has neuroprotective effect, and facial nerve has better protective and nutritional effects.^[32]^ At the same time, Angelica sinensis has the effects of inhibiting smooth muscle contraction, immunomodulation, anti-tumor, anti-radiation, anti-inflammatory, and protecting cardiovascular and cerebrovascular. Like Ligusticum chuanxiong, Angelica sinensis is widely used.^[33]^ Saposhnikovia divaricata has a wide range of cardiovascular benefits, such as antihypertensive, antiatherosclerotic, antiarrhythmic, hypolipidemic, and cardioprotective effects. For patients with facial paralysis, its vascular nutrition and protection are essential for the improvement and recovery of facial paralysis symptoms during convalescence.^[34]^ Astragalus polysaccharide, the active ingredient of Astragalus membranaceus, is an immunopotentiator and regulator, which has antiviral, anti-tumor, anti-aging, anti-radiation, anti-stress, antioxidant, and other activities. For patients with facial paralysis, it can improve immunity, reduce the toxicity of herpes zoster virus, and protect local tissue blood vessels.^[35]^ Angelica dahurica has anti-inflammatory, anti-tumor, antioxidant, analgesic, and other active ingredients, and its antiviral and antibacterial effects have a significant positive impact on the cardiovascular system and the nervous system.^[36]^ As an animal medicine, centipede is considered to be an important drug to promote blood circulation and anticoagulation.^[37]^ White peony root has anti-inflammatory, immunomodulatory, anti-tumor, antiviral, antibacterial, antioxidant, hepatoprotective, and neuroprotective effects.^[38]^

From the pharmacological effects of the above commonly used drugs, it is not difficult to find that traditional Chinese medicine treatment of this disease is mainly through antiviral, antibacterial, improving blood circulation, protecting cardiovascular and cerebrovascular and nerve, and other ways to play a role, the mechanism of a single drug is similar to that of drugs used in modern medicine, but traditional Chinese medicine therapy is not simply the use of a single Chinese medicine, but a variety of drugs. At this time, its active ingredients and mechanism of action are extremely difficult to explore clearly.

## 5. Traditional Chinese medicine treatment of herpes zoster virus infection facial paralysis commonly used therapy

In the acute stage, the disease may be accompanied by pain behind the ear, which is mainly caused by the attack of exogenous pathogens on the collaterals and the accumulation of pathogenic toxins in the interior. At this time, Zhongyi Ointment can be used to relieve the pain. This ointment mainly uses rhubarb, Phellodendron, tangerine peel, Arisaema, magnolia bark, and other drugs to play a role in removing blood stasis and coagulation, so as to achieve the purpose of clearing heat and detoxifying, dissipating blood stasis, and detumescence.^[39]^ In addition, Qianzheng Powder also has a good effect on the treatment of this disease. On the basis of the original prescription, Rhizoma Typhonii, Bombyx Batryticatus, Scorpio, Radix Saposhnikoviae, Radix Gentianae, Radix Isatidis, Flos Lonicerae, Rhizoma Atractylodis, Radix Angelicae Dahuricae, Fructus Forsythiae, and other drugs give full play to their effects of clearing away heat and toxic materials, promoting blood circulation and dredging collaterals, expelling wind and removing.^[40,41]^ In addition, the disease can also be caused by liver depression and qi stagnation due to internal injury of 7 emotions and unsmooth emotions, and is also caused by toxin, and at this time, the modified Chaihu Shugan Powder, namely Bupleurum, Cyperus rotundus, Fructus Aurantii, Radix Paeoniae Rubra, Rhizoma Ligustici Chuanxiong, Pericarpium Citri Reticulatae, Radix Glycyrrhizae, and the like are used together with medicines of Rhizoma Typhonii, Bombyx Batryticatus. It is a good prescription for the treatment of this disease.^[42]^ Fumigating the affected side of the face with traditional Chinese medicine is also a novel idea, such as fumigating the face with the medicinal mist formed by decocting divaricate Saposhnikovia root, Ligusticum wallichii, cassia twig, angelica dahurica, kudzu root, Notopterygium root, stiff silkworm, scorpion, and other medicines, and utilizing the characteristics of easy absorption of the skin to directly reach the focus, so that the effects of promoting blood circulation and removing blood stasis, dredging collat.^[43]^ For patients in acute stage, Daqin Decoction can also be used to play its role in expelling wind, dredging collaterals and removing arthralgia, and the curative effect is also good,^[44]^ while Daqin Decoction has good pharmacological effects of anti-inflammatory, analgesic, and enhancing immune function.^[45]^ Decoction for Warming the Middle and Dispelling Cold has a miraculous effect in the treatment of acute facial paralysis. For patients with deficiency of vital qi and imbalance of yin and Yang, Ligusticum chuanxiong and Angelica sinensis have the effects of promoting blood circulation, removing blood stasis, nourishing blood, and activating blood circulation, which can effectively improve facial dysfunction.^[46]^

It can be seen that a variety of traditional Chinese medicine combined with treatment can complement each other, act on the human body through a variety of ways, and directly reach the lesion. The curative effect is far better than a single herb treatment. However, herbs have toxic and side effects and cannot be used for a long time. Acupuncture is needed to replace long-term treatment to achieve better efficacy.

## 6. The feasibility of acupuncture combined with medicine therapy in the treatment of herpes zoster virus infectious facial paralysis

From the above, it can be found that traditional Chinese medicine treatment of this disease has a good effect, acupuncture therapy has the advantage of rapid effect and no adverse reactions, while traditional Chinese medicine therapy can give patients lasting and stable treatment by oral or external use. So, can the 2 be combined to improve the symptoms of facial paralysis in a timely, lasting and stable manner until they are cured?

**Figure 1.**
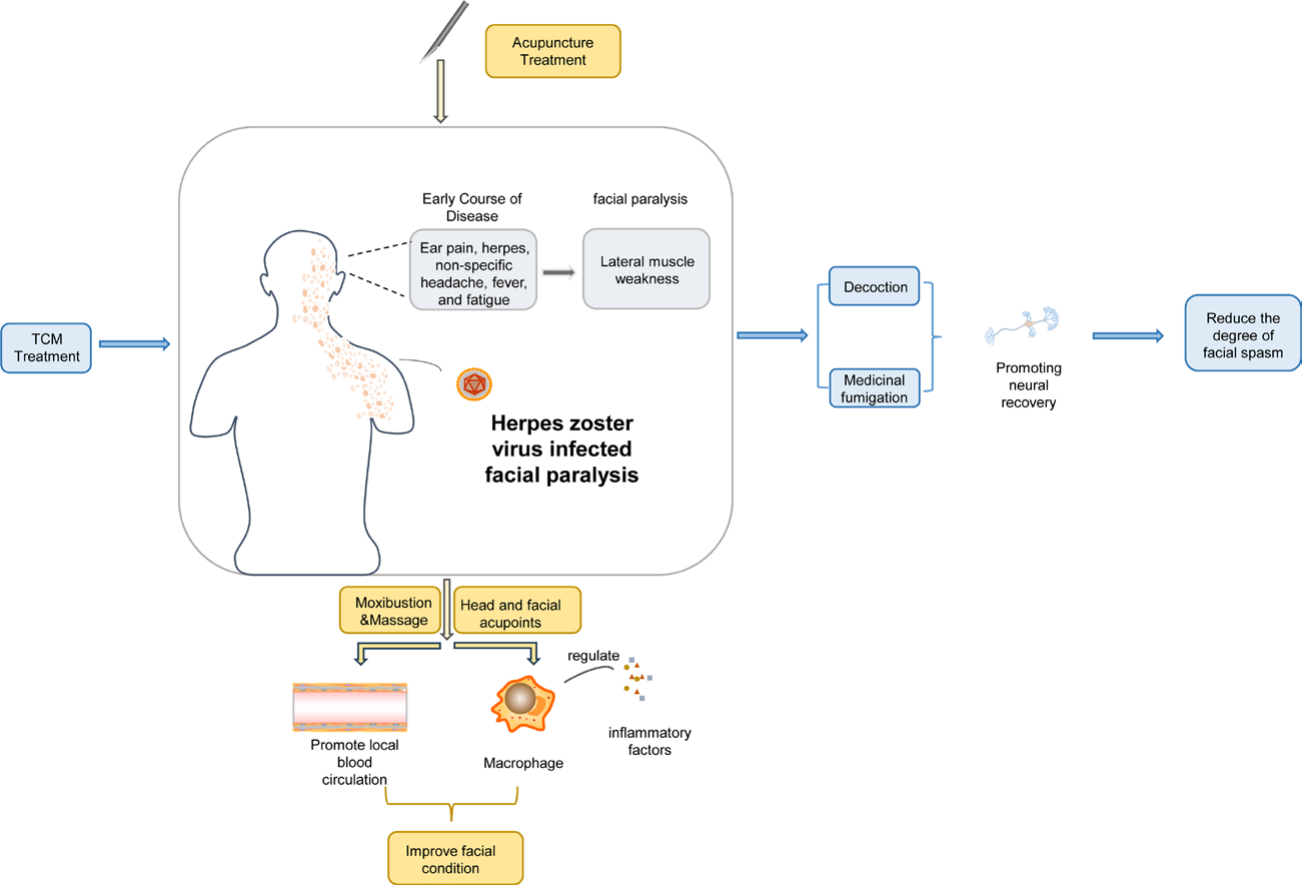
The mechanism of acupuncture combined with traditional Chinese medicine in the treatment of herpes zoster infectious facial paralysis.

Studies have found that the combination of acupuncture and medicine in the treatment of various types of facial paralysis has a very perfect complementary effect, that is, acupuncture with medicine can increase the sustainability of treatment, and acupuncture with medicine can make the treatment more rapid and direct.^[47]^ For example, Sheng Canruo takes 3 needles on the face (1 cun below the ground storehouse, penetrating the cheek car; 2: the big face penetrates the thorn to the cheekbone; 3: 1 cun below the sun, penetrating acupuncture to Sibai) mild stimulation combined with oral administration of traditional Chinese medicine compound Qianzheng powder, the effect of internal and external treatment is remarkable.^[48]^ The total effective rate of facial paralysis treated by Qianzheng powder and Taohong Siwu Decoction combined with acupuncture was significantly increased.^[49]^ In addition, in the acute stage of the disease, the combined treatment of filiform acupuncture at the acupoints of the affected part and facial paralysis powder (composed of Geranium wilfordii, Ephedra, Pueraria lobata, Astragalus membranaceus, Saposhnikovia divaricata, Schizonepeta tenuifolia, Angelica sinensis, Typhonium giganteum, Scorpio, Bombyx Batryticatus, Periostracum Cicadae, Ligusticum wallichii, Ramulus Cinnamomi Thereby relieving symptoms).^[50]^ In the recovery period, Buyang Huanwu Decoction plus Qianzheng Powder combined with acupuncture is used to treat facial paralysis patients with deficiency of qi and blood and accumulation of qi, phlegm, and blood stasis, which is very significant compared with Western medicine or simple acupuncture or traditional Chinese medicine treatment.^[51]^ Some special treatments such as electroacupuncture and surface stimulation of plum blossom hammer and traditional Chinese medicine, combined with conventional treatment, can also achieve better results in the treatment of this disease,^[3]^ external application of traditional Chinese medicine combined with acupuncture treatment on the affected acupoints has a faster recovery effect on the facial paralysis muscles in the acute stage^[52,53]^(Fig. 1).

From the above discussion, it is not difficult to find that the combined therapy of acupuncture and medicine has achieved quite good results in clinical practice, but there are still some problems-lack of experimental data to verify and support the correctness of the conclusion, clinical results may be probabilistic events, not feasible, and so on. These are the key areas for future research.

## 7. Discussion

After herpes zoster virus invaded the human body for the first time, although the symptoms of herpes were cured after treatment, the herpes virus was still latent in the human body. When the immunity of the human body was low and the healthy qi was insufficient, it would recur and infect the geniculate ganglion, thus causing the dysfunction of the nerve and below. The main clinical manifestations were facial paralysis and earache. In the early stage of discovery, timely antiviral treatment can effectively prevent the deterioration of the disease, but patients often get the wrong treatment because of some symptoms such as fever and herpes in the early stage of the disease, and only use simple drugs for fever or herpes, so the disease is treated by mistake and develops into facial paralysis, and because of herpes virus. Therefore, compared with other peripheral facial paralysis, the treatment of this disease is more difficult. Western modern medicine has always adopted the program of anti-virus combined with nerve nutrition, but this program is suitable for the acute or early stage of this disease, and has no obvious effect on the treatment of later and recovery stages. Traditional Chinese medicine treatment of this disease to improve symptoms as the main purpose, acupuncture and moxibustion treatment can stimulate the nerve, improve local blood circulation, and then gradually alleviate the symptoms of facial paralysis, in addition, the comprehensive treatment of traditional Chinese medicine, whether oral or external use, has antibacterial and antiviral effects to improve immunity, the combination of the 2 treatment can not only be more effective, but also more lasting and stable. However, in the pursuit of improving symptoms, the mechanism of action of the 2 is not clear, and there is a lack of sufficient data support, which is also the next key research direction.

However, there are still some challenges in the treatment of acupuncture combined with traditional Chinese medicine. Although the curative effect is supported by clinical cases, the internal mechanism of treatment is still unclear. International acceptance of traditional Chinese medicine is also a challenge. But this is also an opportunity to overcome these difficulties after the worldwide patients can get better treatment. Similarly, researchers and medical practitioners will also be more respected, and the opportunities will gradually increase.

## Author contributions

**Data curation:** Ruiqian Guan.

**Investigation:** Limin Pan.

**Methodology:** Yusu Wang.

**Software:** Yeyao Li.

**Writing – original draft:** Zhibo Hong.

**Writing – review & editing:** Zhibo Hong.
